# Septic shock and the use of norepinephrine in an intermediate care unit: Mortality and adverse events

**DOI:** 10.1371/journal.pone.0183073

**Published:** 2017-08-24

**Authors:** Mikael Hallengren, Per Åstrand, Staffan Eksborg, Hans Barle, Claes Frostell

**Affiliations:** 1 Department of Clinical Sciences, Division of Anaesthesia and Intensive Care, Karolinska Institutet, Danderyd Hospital, Stockholm, Sweden; 2 Department of Clinical Sciences. Division of Internal Medicine, Karolinska Institutet, Danderyd Hospital, Stockholm, Sweden; 3 Department of Women's and Children's Health, Childhood Cancer Research Unit Q6:05 Karolinska Institutet, Astrid Lindgren Children's Hospital, Karolinska University Hospital Solna, Stockholm, Sweden; 4 Claes Frostell Research and Consulting AB, Stockholm, Sweden; Azienda Ospedaliero Universitaria Careggi, ITALY

## Abstract

**Background:**

Septic shock is associated with high mortality. Aged and multimorbid patients are not always eligible for intensive care units. Norepinephrine is an accepted treatment for hypotension in septic shock. It is unknown whether norepinephrine has a place in treatment outside an intensive care unit and when given peripherally.

**Objectives:**

To describe mortality, Acute Physiology And Chronic Health Evaluation (APACHE-II), time to mean arterial pressure >65 mmHg, and adverse events in patients with septic shock receiving norepinephrine peripherally in an intermediate care unit.

**Methods:**

From a retrospective chart review of 91 patients with septic shock treated with norepinephrine for hypotension, ward mortality, 30-, 60- and 90-day mortality, standardized mortality ratio (SMR) and adverse events (necrosis and arrhythmia) were analysed. Administration route via peripheral venous catheter or central venous catheter was registered.

**Results:**

Median age was 81 (43–96) years and median APACHE-II score was 26 (12–42). Observed ward mortality was 27.5% (SMR 0.443, 95% CI: 0.287–0.654), and 30-day and 90-day mortality were 47.2% and 58.2%, respectively.

**Conclusions:**

Elderly patients with septic shock treated with norepinephrine displayed a better survival in the ward and at 30 days than expected. Our retrospective study did not indicate frequent complications when administering norepinephrine via a peripheral venous catheter.

## Introduction

The measurement of systemic arterial pressure by mean arterial pressure (MAP) or systolic plus diastolic pressures (SAP, DAP) has been considered an appropriate way to achieve meaningful clinical information for more than a century. By measuring blood pressure, systemic hypertension has been identified and treated for years. It was noted that the development of hypotension associated with severe infection singled out patients with obviously worse prognosis. Thus, the distinction between “sepsis” and “septic shock” was established in 1992 [1]. In Sweden 210 per 100,000 people had sepsis, and 30 per 100,000 developed septic shock in 2012 [2]. In that same year, 1,000 patients died from sepsis according to the Cause of Death Register established by the Swedish National Board of Health and Welfare [3]. The ICU mortality in Sweden was 34% in 2012 according to the Intensive Care Unit Register Sweden [4].

In many trials and clinical materials, septic shock is defined as present when SAP is below 90 mmHg or MAP is below 65 mmHg, despite an intravenous (IV) fluid bolus [1, 5–7]. Patients with septic shock are typically treated in an intensive care unit (ICU). They receive an IV fluid bolus, often through a central venous catheter (CVC) that has been placed in addition to a peripheral venous catheter (PVC). Then, they are continuously monitored with pulse oximetry and repeated controls of blood pressure and heart rate, diuresis, breathing and consciousness. If hypotension remains in spite of aggressive fluid administration, an infusion of a vasopressor like norepinephrine (NE) is usually started in a CVC with the aim to reach and maintain a MAP of at least 65 mmHg [5].

In 2001 Rivers et al. presented the concept of early goal directed therapy (EGDT) for severe sepsis and septic shock [6]. Lately, several studies have evaluated EGDT and found that EGDT is not superior compared to standard care [8–10]. Although standard care maybe more like EGDT now than prior to 2001, these studies suggest that extensive monitoring such as cardiac output via pulmonary artery catheter, SvO2 or central venous pressure in these patients may not be necessary.

ICU-resources often are stretched markedly thin in a modern busy city hospital. Many patients tend to be quite old (>80 years of age), displaying multiple organ deficiencies even before the onset of an acute illness with infection and hypotension. As a result of previously agreed-upon treatment limitations and general overcrowding, acutely ill patients may be stranded outside an ICU in emergency care or intermediate care facilities or elsewhere into the hospital system.

We were recently made aware of a local treatment algorithm established at the Intermediate Care Unit (IMCU), Division of Internal Medicine, Danderyd Hospital outside Stockholm, where patients with sepsis and hypotension were offered vasopressor support for up to 72 hours outside the ICU. Prior to treatment in the IMCU, a discussion regarding the patient’s care was held with an intensivist, resulting in the decision to admit and treat the patient in the IMCU rather than in the ICU. The patients were placed in an intermediate care area with a nurse to patient ratio of at least 1:3. Then, they received IV fluids and an NE infusion and were monitored for possible arrhythmias with telemetry. This paper is a retrospective report describing the included patients, clinical outcomes, and observed complications.

## Materials and methods

### Clinical setting and subjects

The study was conducted as a retrospective survey at the Intermediate Care Unit (IMCU), Division of Internal Medicine, Danderyd Hospital, Stockholm, Sweden. The IMCU holds six beds and a minimum nurse to patient ratio of 1:3. The nurses interacted with an experienced internal medicine resident with support from a senior consultant. However, no intensivist was present or on call at the IMCU.

**Fig 1:**
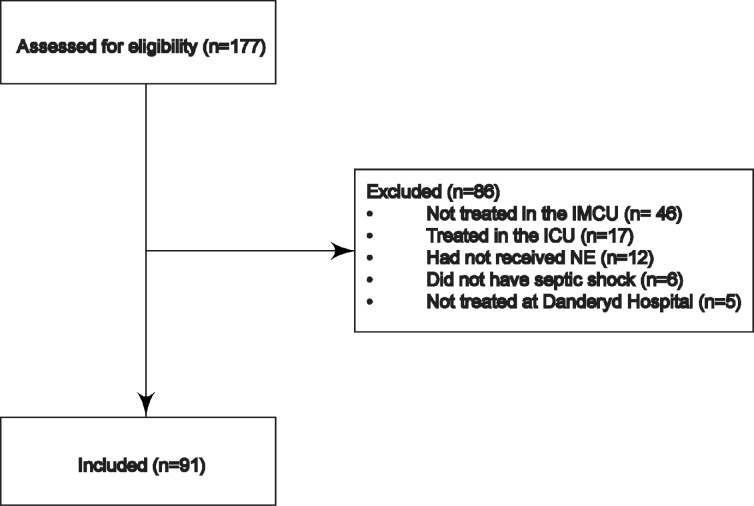
Consort chart. Consort chart showing patients eligible for the study and reason for exclusion. IMCU = Intermediate Care Unit, ICU = Intensive Care Unit, NE = Norepinephrine.

Using the medical charts, we screened all patients who had received NE at Danderyd Hospital and included patients treated in the IMCU for septic shock. Inclusion criteria for study entry were age ≥18 years, meeting the definition of septic shock, subsequent administration with an infusion of NE, and treatment in the IMCU. Patients were excluded if they were treated in the ICU during the same period of hospitalization (Fig 1).

### Patient selection

We identified 177 patients between 2011 and 2014 by searching Danderyd Hospitals’ computerized medical charts for “norepinephrine” using QlikView® (QlikTech International, Lund, Sweden) and extracted data from the electronic chart TakeCare® (CompuGroup Medical, Stockholm, Sweden). Data were obtained through the patients’ social security number and were not anonymous. A search for the ICD-10 code R.57.2 *Septic shock* was made; however, it became apparent that a majority of patients were coded incorrectly. Instead, all patients who were prescribed NE were identified, with the exception of patients treated in the ICU, which uses another non-computerized patient chart. A total of 91 patients were included in this study.

### Data collection

From all included patient data, age, gender, and parameters for Acute Physiology and Chronic Health Evaluation II (APACHE-II) [11], and the KDIGO acute kidney injury classification system were collected [12]. Data were extracted from TakeCare regarding IMCU-mortality, mortality up to 90 days from admission, IMCU length-of-stay (LOS), NE administration through a PVC or a CVC, and expected complications (skin necrosis or pale skin around the PVC and arrhythmias). Patients admitted to the hospital before the onset of septic shock were identified. These patients had a time documented in TakeCare when MAP <65 mmHg was observed. Thus, the time from identifying MAP <65 mmHg to the start of NE infusion as well as to restoration of adequate MAP (>65 mmHg) could be calculated. Each participant’s dataset was coded with a number matching the social security number; the key is held by the first author.

### Principles of care and management goals

#### Monitoring

According to an established local protocol for NE use in the IMCU at Danderyd Hospital, the patients were continuously monitored with heart rate sensors, three-point ECG, non-invasive blood pressure (NIBP) every 5–15 minutes, and continuous pulse oximetry. The ECG was monitored through telemetry, which allowed recorded events to be viewed retrospectively. Point-of-care arterial blood gas was taken at admission and as needed according to local clinical routines.

#### Antibiotics

All patients received broad-spectrum antibiotics until cultures and sensitivity tests were done. Antibiotics were modified according to the susceptibility test.

#### Blood pressure goals

The goal of treatment was a MAP from 65–70 mmHg. If the patient did not respond with a MAP >65 mmHg after a fluid bolus of 500–1000 ml Ringer’s acetate, treatment with NE was initiated with 0.05 microg/kg/min. The infusion rate was titrated up if MAP <65 mmHg and down when MAP >70 mmHg. The highest infusion rate allowed (according to the local protocol) in the IMCU was 0.2 microg/kg/min. Fluid treatment was prescribed according to the individual physician’s clinical assessment of the patient’s grade of dehydration.

#### Period of hypotension

It would have been optimal if the onset of septic shock and hypotension had been known in all patients in a study of this kind. However, many patients fell ill at a nursing home or in their home where no blood pressure was measured at the onset of symptoms. In addition, several patients were sent from an ordinary hospital ward where blood pressures usually are measured three times a day or at the onset of clinical deterioration. Hence, the exact time-point for the debut of hypotension in most patients remains undetermined in this study.

#### Blood tests

All blood test analyses required for calculating APACHE-II were performed by Karolinska University Laboratory, which had been previously accredited by ISO 15189 “Requirements for quality and competence.” Point-of-care testing for arterial blood gases has no accreditation.

### Calculating MAP

Since NIBP gives systolic blood pressure (SAP) over diastolic blood pressure (DAP), a calculation of MAP was necessary. MAP was calculated through the formula MAP = DAP + (SAP—DAP) / 3 [13].

### Statistical analysis

Standardized mortality ratio (SMR) and its 95% confidence intervals were calculated as given by Liddell [14]. The meaning and use of the Receiver Operating Characteristic (ROC) curve is given in Kumar et al. and Hanley et al. [15, 16]. MS Excel (Microsoft Corporation, Redmond, Washington, USA) was used for data collection; GraphPad Prism version 5.04 (GraphPad Software Inc. San Diego, USA) was used for statistical analysis. P-values <0.05 were considered statistically significant. Reported p-values are from two-sided tests.

### Ethical approval

Ethical approval was received from Stockholm’s Regional Ethics Board (2013/1537-31/4). Because the study is a retrospective survey, no written consent was possible to obtain from the participants and was not needed according to the ethical approval requirements.

## Results

### Patient characteristics

The patient characteristics are presented in Table 1.

**Table 1:** Patient characteristics. Median and range or numbers and percent.

Factor		
Age	Years	81 (43–96)
Sex	Female n (%)	35 (43.2%)
	Male n (%)	46 (56.8%)
Body weight	kg	65 (37–140)
Body Mass Index	kg/m^2^	21.8 (13.6–44.1)
MAP start of treatment	mmHg	56 (35–65)
Crystalloid fluids hour 1	ml	1000 (<500–3000)
Crystalloid fluid day 1	ml	5000 (500–9100)
Days admitted (IMCU)	Day started	3 (1–22)
APACHE-II	Score	26 (12–42)
KDIGO AKI Classification	Score	1 (0–3)
Patients with at least 1 KDIGO AKI Classification		77 (84.6%)

AKI = Acute kidney injury, APACHE = Acute physiology and chronic health evaluation, IMCU = Intermediate Care Unit, KDIGO = Kidney disease improving global outcome, MAP = mean arterial pressure.

### APACHE-II-score and mortality

**Fig 2:**
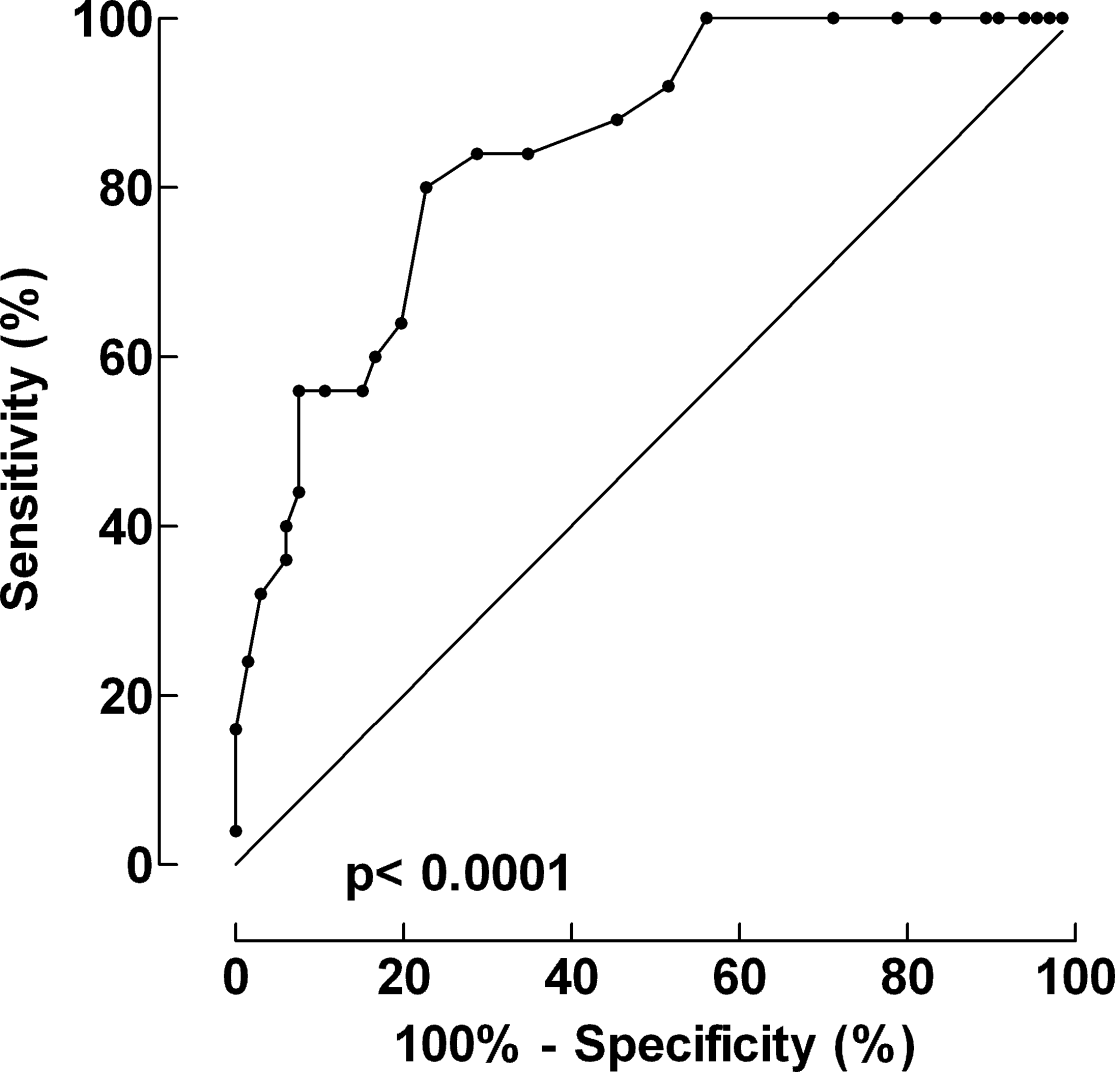
ROC-analysis for the specificity and sensitivity of APACHE-II. ROC = Receiving operating characteristics. APACHE = Acute physiology and chronic health evaluation.

The median APACHE-II-score was 26 (12–42), translating to an estimated mortality rate (EMR) of 59.7% (16.1–93.9%). Observed IMCU mortality was 27.5% (SMR 0.443, 95% CI: 0.287–0.654), and 30-day and 90-day mortality were 47.2% and 58.2%, respectively. Fig 2 shows that APACHE-II accurately described mortality outcome in our material.

### ROC analysis

The results of the ROC analysis are given in Fig 2. The area under the curve AUC was 0.85 (95% CI: 0.76 to 0.93), p<0.0001.

### Blood pressure goals

**Fig 3:**
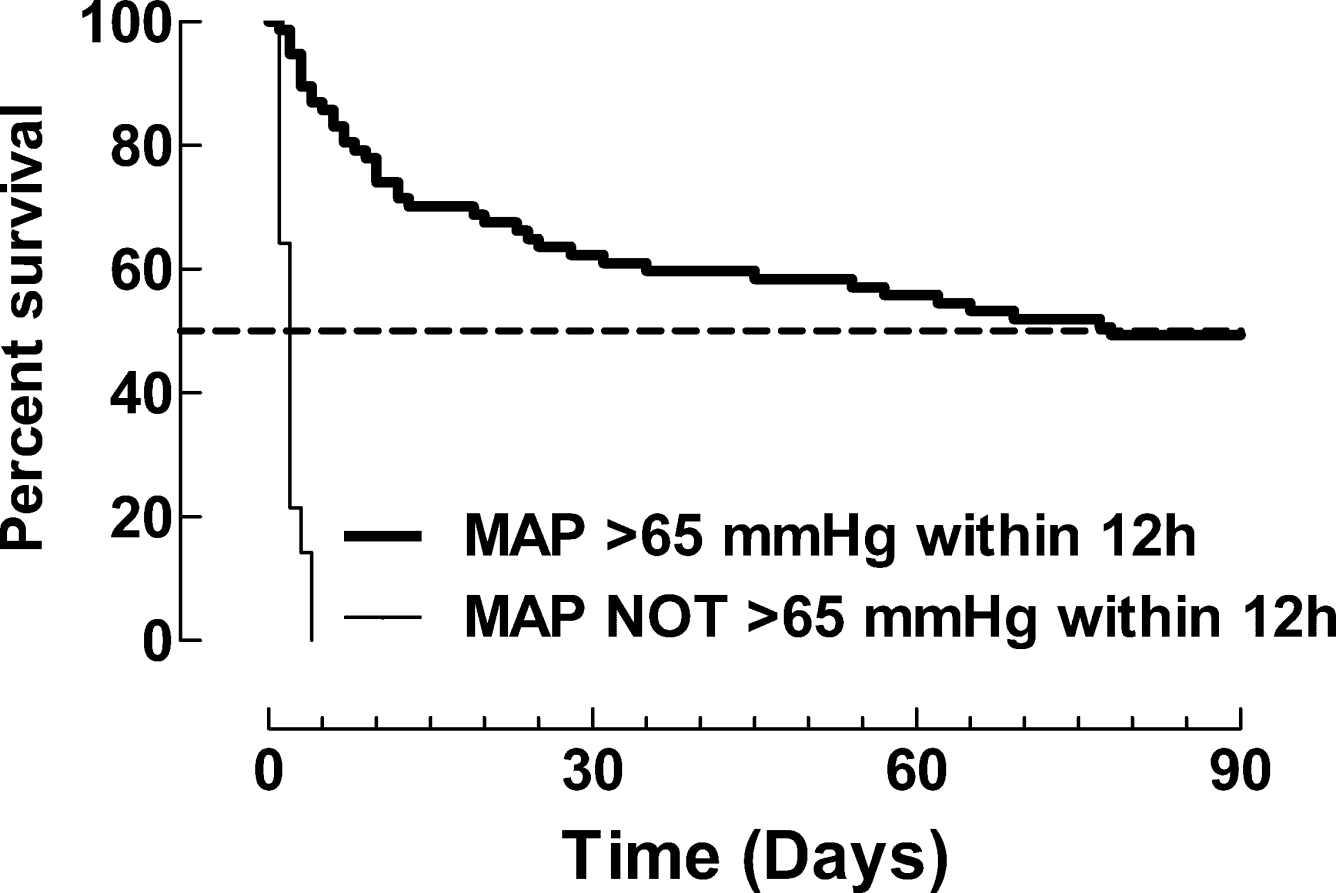
Mortality (%) for patients achieving versus not achieving MAP >65 mmHg within 12 hours. Kaplan-Meier curve with mortality (%) as outcome for patients achieving versus not achieving MAP >65 mmHg within 12 hours. p<0.0001 (Gehan-Breslow-Wilcoxon Test).

Patients not achieving a MAP >65 mmHg in 12 hours displayed poor prognosis (Fig 3).

**Fig 4:**
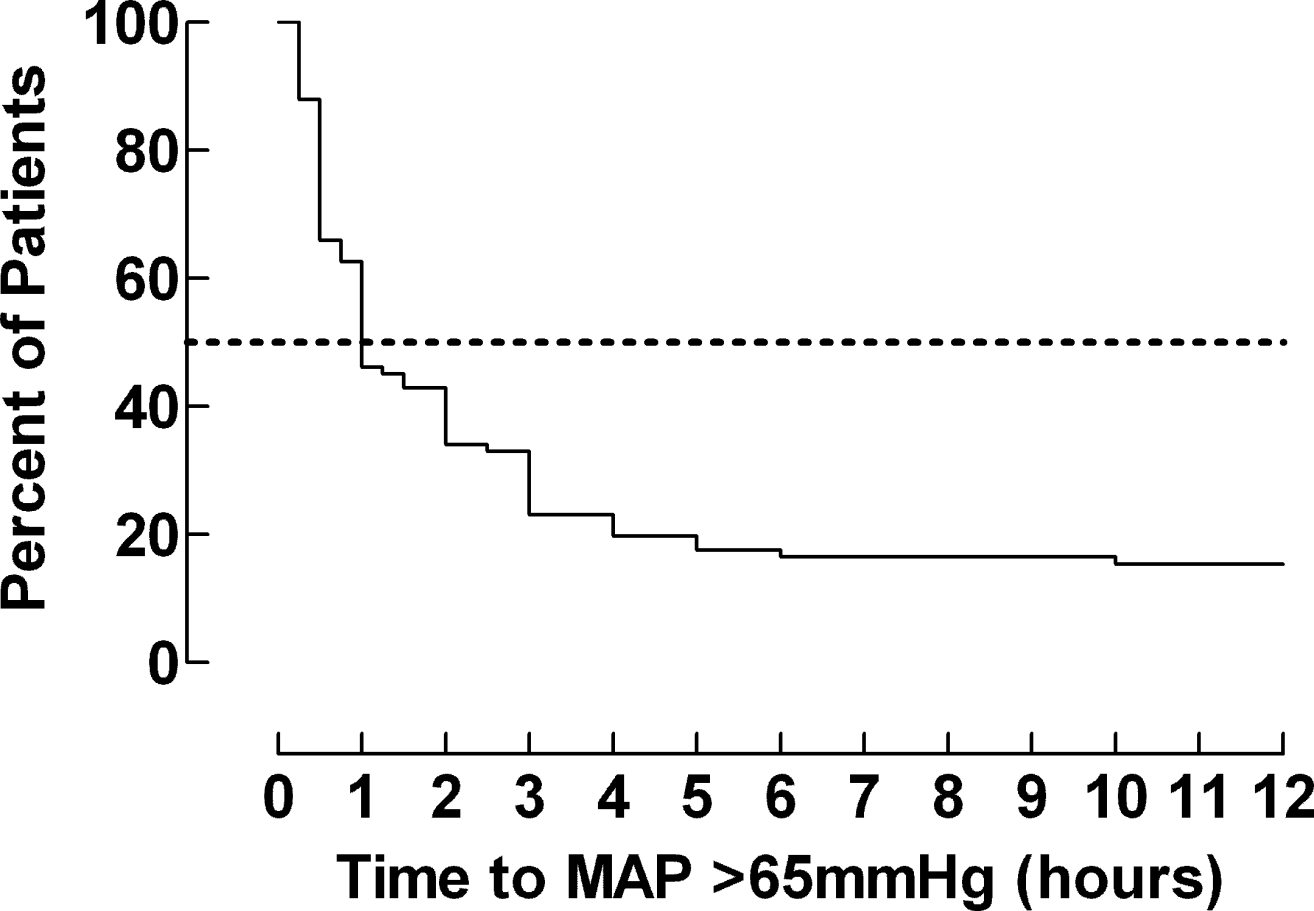
Kaplan-Meier curve describing time to MAP > 65mmHg.

Most patients achieving a MAP >65 mmHg did so within 1 hour (0.25–10) from the start of the NE infusion (Fig 4).

### Observed blood pressure fall

**Fig 5:**
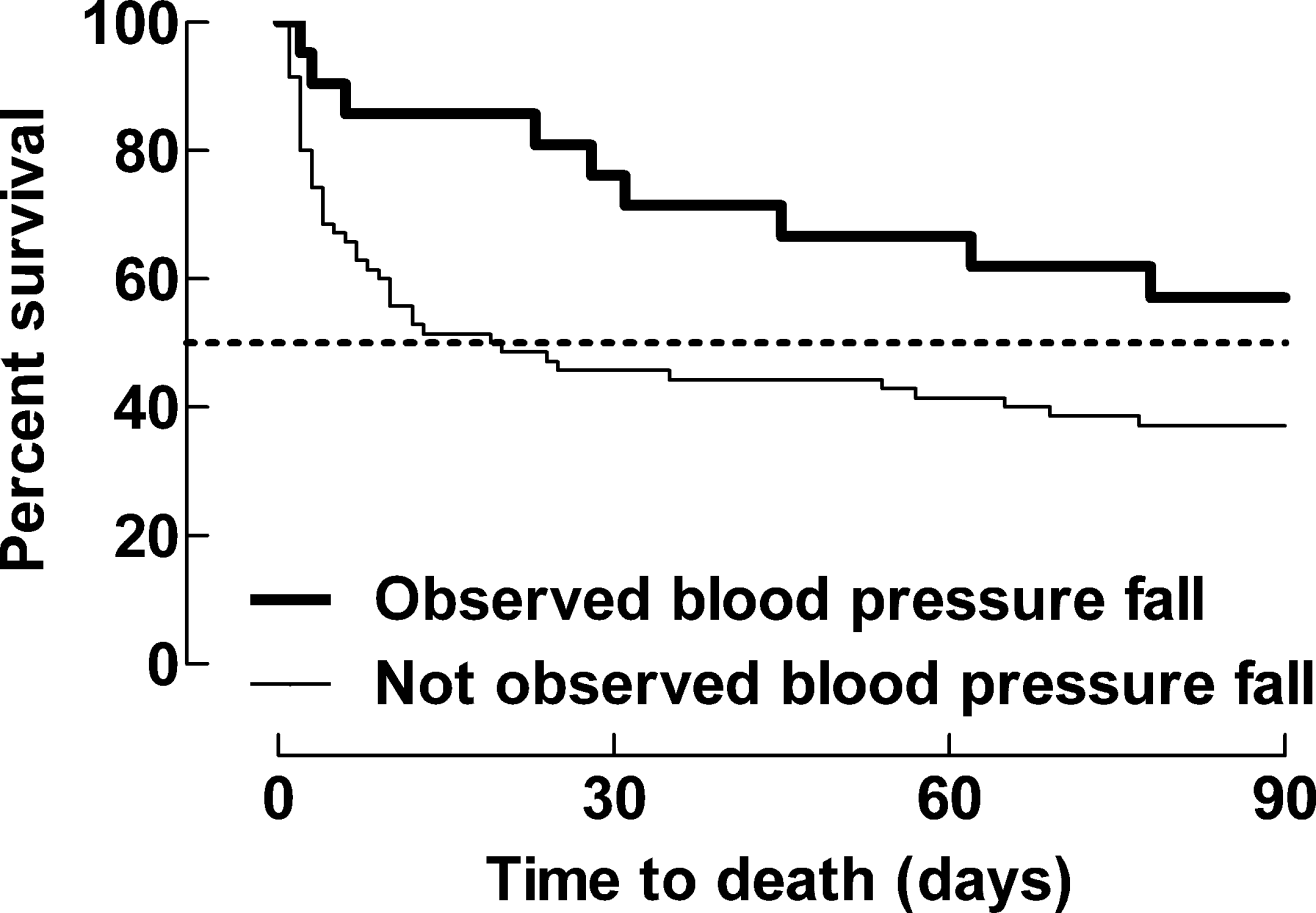
Kaplan-Meier curve comparing patient mortality with point of time was known or unknown for a blood pressure fall below MAP 65 mmHg. p = 0.0291 (Gehan-Breslow-Wilcoxon Test).

In 23% of the patients (n = 21), the timing for a fall in blood pressure <65 mmHg could be established. Mortality was significantly lower in this patient group compared to patients where the time of blood pressure fall could not be established (Fig 5). The IMCU mortality was 14.3% (n = 3), and the 90-day mortality was 42.8% (n = 9). Their median time to NE infusion was 1.5 hours (1–6 hours) from admission.

### Complications

Through notes in the charts, we deduced that no patient showed any signs of ischemia or necrosis around the area of infusion. One patient had sinus tachycardia up to 180 beats per minute at NE infusion rates above 0.09 microg/min/kg and never achieved MAP >65 mmHg. The NE infusion had to be discontinued, and the patient subsequently died in the IMCU within a few hours. Two patients had PVCs that did not work due to high resistance alarms from the infusion pumps. One patient had the PVC changed, and one received a CVC. Finally, one patient required three attempts to secure venous access at the neck until a CVC was established, with a resulting large hematoma close to the site of insertion.

### PVC or CVC

In the majority of patients (n = 79; 86.8%), the NE infusion was administered through a PVC. The remaining patients had a CVC (n = 12; 13.2%).

### Duration of therapy with NE

The median time for NE infusion was 13 hours (range 0.5–72 hours). Blood pressure was monitored every 5 minutes when MAP <65 mmHg and every 15 minutes when MAP >65 mmHg. However, it was not noted in the charts every time a new blood pressure was measured; hence, the data for the time to MAP >65 mmHg are probably underestimated.

## Discussion

We have conducted a retrospective chart study to identify 91 mostly elderly hospitalized patients with multiple morbidities suffering from septic shock and not considered eligible for ICU admission. Our main and surprising finding was that subsequent treatment with NE in the IMCU resulted in an IMCU mortality of only 27.5% (SMR 0.443), with a 30-day survival of 52.8%. The vast majority of patients had a peripheral venous catheter (PVC) as the only venous access with few complications—most importantly, no incidence of skin necrosis.

### Material and mortality

The median APACHE-II score was 26, and the median age was 81 years in this retrospective study. In other studies, patients with septic shock [17–19] with a respective mean APACHE-II score of 22, 20 and 24 had reported a median age of 57, 54 and 32 years, respectively. The higher APACHE-II score in this study could partly be explained by the advanced age of the included patients. No studies presenting mortality rates in patients with septic shock who were treated at an IMCU have been found by the authors. Two previous studies focusing on patients with septic shock in the United States found an ICU mortality of 40% and a hospital mortality of 50% [20, 21]. The ICU mortality in Sweden for R57.2 Septic shock was 34% in 2012 [4]. A large, multicentre study from France that included 1,488 patients with septic shock reported an ICU mortality of 40% and a 90-day-mortality of 52% [22]. The relatively low mortality in our material may be explained partially by effective hospital logistics, where patients are moved quickly from the emergency room to the IMCU for treatment. In addition, no patients in this study needed concomitant invasive ventilation support of any kind.

### ROC analysis and use of APACHE II

As demonstrated in Fig 2, ROC analyses with a high AUC value and a low p-value show a high internal validity of APACHE II as predictor of mortality in this limited group of 91 individuals. The use of the ROC curve indicates an optimal cut-off value for an APACHE-II score of 68.05, giving a sensitivity of 80% and a specificity of 77% [15]. APACHE-II is well validated and has been used widely for several years to describe the severity of disease [11].

### Definition of sepsis

This study used the same definition of septic shock as is described in the Surviving Sepsis Campaign 2012: septic shock involves hypotension despite fluid resuscitation in a patient with severe sepsis [5]. Recently, Singer et al (2016) published a suggestion for a new definition of sepsis and septic shock [23]. We have decided to use the older definitions for a few reasons. Firstly, these data collections were finished when the new definitions came out. Secondly, the new definitions have yet to be reviewed by the intensive care society.

Only a minority (n = 18) of patients had the diagnosis of R57.2 Septic Shock in their charts. Therefore, we used the term”norepinephrine” as a proxy for identifying patients with septic shock. It is disturbing for future register studies and retrospective studies that so few patient charts had the correct diagnosis listed.

### Blood pressure goal

In a minority of patients (n = 21), it was possible to establish the approximate point of time at which MAP fell below 65 mmHg. Interestingly, this subgroup of patients had a lower mortality in comparison with the remaining patients where time of onset of hypotension was less clear. Data from previous studies [24, 25] suggest that early versus delayed treatment with NE gives more benefit; this observation that has greatly influenced therapy tradition in the Western practice. We suggest that one reason for the lower mortality in the patients with a known onset of hypotension was the relatively quick initiation of effective therapy with NE in the IMCU (median time 1.5 hours from detection). In contrast, patients who never reached a MAP >65 mm Hg, in spite of fluid and low-dose NE, had an extremely poor prognosis and died within 72 hours of the start of treatment. In a future study, we suggest it would be of interest to follow blood lactate throughout the treatment period of septic shock.

We are painfully aware of the fact that ***systemic blood pressure*** is a poor proxy parameter for global perfusion. Perhaps cardiac output, or other more precisely quantifiable variables in blood or tissue could describe the shock state better [26–28]. However, MAP or systolic blood pressure remains a vital part of the clinical detection and definition of the borderline between “sepsis” and “septic shock”, and it is the parameter available in the emergency room or at a general ward as a sign of alarm once clinical deterioration becomes obvious. In future research, we may be able to agree upon and validate a pattern of clinical signs and blood sampling into a better-defined classification of states of shock and the recovery process from shock.

### Venous access

NE is a potent vasoactive agent, and the general recommendation is to administer NE via a CVC [5]. We have found one review of 85 papers with 270 patients reporting about local tissue injury when vasopressors were administered in a peripheral vein. Loubani et al. [29] noted 325 extravasation events in these patients. Most of the studies included in this review are case reports, which make it difficult to assess the risk (incidence) for such an adverse event. In our study, we examined charts from 79 patients treated with NE in a PVC for 0.5 to 72 hours and found none who had this complication reported. Recently, other descriptive, retrospective studies in a pediatric setting have also demonstrated some safety in administering NE in a PVC during resuscitation or transport [30, 31]. It is not completely safe to insert a CVC due to fairly common complications such as cardiac arrhythmias, accidental arterial punctures, and hematomas at the place of catheter insertion [32]. One randomized trial showed that inserting a CVC in the ICU resulted in fewer complications than inserting a PVC. Complications considered major were erythema around the point of insertion and difficulties inserting and maintaining the catheter [33]. The lack of larger randomized trials focused on safety aspects in administering vasopressors in either a PVC or CVC are obvious, and we would welcome such future work.

### Ethical issues, limitations of therapy and prognosis

Clearly, several factors represent significant ethical, logistical, and practical challenges for patients, relatives, and hospital staff in a geriatric hospital population when sepsis develops. Among these, we recognize previous decisions regarding limitations of treatment, a generally poorer prognosis due to concomitant illness, and the overcrowding of many hospitals. Our patients had a median age of 81 years, and 85% either had previous kidney insufficiency or developed AKI as a result of their acute illness. Thus, they represent one of the most ill and compromised patient cohorts in the hospital at any moment. We argue it is therefore possible to identify causes for some therapeutic nihilism that threatens to influence choice of therapy for such patients. Such behaviour, however, should not remain excusable. These patients do not easily compete for scarce hospital resources. In balance, we argue that the chosen treatment strategy was ethically sound and could be defended, that retrospective outcome data are encouraging, and that serious complications were few. Only a future randomized study can define the true patient benefit of applying this type of treatment bundle in a septic insult with hypotension in a geriatric hospital population.

Judging from data on SMR, derived from an APACHE-II estimated mortality ratio (EMR) per patient, and using mortality in the IMCU as proxy for ICU mortality, it appears reasonable to state that short-term (IMCU and 30-days) prognosis was not seriously compromised by treating a group of patients according to this alternative algorithm at the IMCU in Danderyd Hospital.

### Study limitations

A few patient charts did not give sufficient information regarding the amounts of fluids, and the blood pressure was not always noted frequently. Staff adherence to agreed-upon treatment protocol was sometimes limited. All data were gathered retrospectively and at a single center. Our conclusions are weakened by the fact that no control group exists in this report, and only retrospective observational data are presently available.

## Conclusion

Elderly patients with septic shock treated with norepinephrine (NE) displayed a better IMCU survival at the ward and at 30 days than previously expected. Our retrospective chart study did not indicate frequent complications when administering NE via PVC.

## Supporting information

S1 File Database with minimal dataset. (XLSX)

S2 File Letter from Karolinska Institute regarding Bench Fee. (PDF)

## References

[1] Angus DC, van der Poll T. Severe sepsis and septic shock. N Engl J Med. 2013;369(9):840–51. doi: 10.1056/NEJMra1208623 23984731 10.1056/NEJMra1208623

[2] Nationella vårdprogrammet för svår sepsis och septisk chock. Uppsala; 2012.

[3] Dödsorsaker 2012. www.socialstyrelsen.se: Socialstyrelsen; 2013.

[4] Stockholm. Sweden ICUR. http://icuregswe.org/utdata/: Intensive Care Unit Register Sweden 2012.

[5] Dellinger RP, Levy MM, Rhodes A, Annane D, Gerlach H, Opal SM, et al Surviving Sepsis Campaign: international guidelines for management of severe sepsis and septic shock, 2012. Intensive Care Med. 2013;39(2):165–228. doi: 10.1007/s00134-012-2769-8 23361625 10.1007/s00134-012-2769-8 PMC7095153

[6] Rivers E, Nguyen B, Havstad S, Ressler J, Muzzin A, Knoblich B, et al Early goal-directed therapy in the treatment of severe sepsis and septic shock. N Engl J Med. 2001;345(19):1368–77. doi: 10.1056/NEJMoa010307 11794169 10.1056/NEJMoa010307

[7] Klassifikation av sepsis, svår sepsis och septisk chock www.socialstyrelsen.se: Socialstyrelsen; 2013 [

[8] Peake SL, Delaney A, Bailey M, Bellomo R, Cameron PA, Cooper DJ, et al Goal-directed resuscitation for patients with early septic shock. N Engl J Med. 2014;371(16):1496–506. doi: 10.1056/NEJMoa1404380 25272316 10.1056/NEJMoa1404380

[9] Yealy DM, Kellum JA, Huang DT, Barnato AE, Weissfeld LA, Pike F, et al A randomized trial of protocol-based care for early septic shock. N Engl J Med. 2014;370(18):1683–93. doi: 10.1056/NEJMoa1401602 24635773 10.1056/NEJMoa1401602 PMC4101700

[10] Mouncey PR, Osborn TM, Power GS, Harrison DA, Sadique MZ, Grieve RD, et al Trial of early, goal-directed resuscitation for septic shock. N Engl J Med. 2015;372(14):1301–11. doi: 10.1056/NEJMoa1500896 25776532 10.1056/NEJMoa1500896

[11] Knaus WA, Draper EA, Wagner DP, Zimmerman JE. APACHE II: a severity of disease classification system. Crit Care Med. 1985;13(10):818–29. 3928249

[12] Group. KDIGOKAKIW. KDIGO Clinical Practice Guideline for Acute Kidney Injury. Kidney Inter., Suppl.2012. p. 1–138.

[13] Rogers G, Oosthuyse T. A comparison of the indirect estimate of mean arterial pressure calculated by the conventional equation and calculated to compensate for a change in heart rate. Int J Sports Med. 2000;21(2):90–5. doi: 10.1055/s-2000-8865 10727067 10.1055/s-2000-8865

[14] Liddell FD. Simple exact analysis of the standardised mortality ratio. J Epidemiol Community Health. 1984;38(1):85–8. 6707569 10.1136/jech.38.1.85 PMC1052324

[15] Kumar R, Indrayan A. Receiver operating characteristic (ROC) curve for medical researchers. Indian Pediatr. 2011;48(4):277–87. 21532099 10.1007/s13312-011-0055-4

[16] Hanley JA, McNeil BJ. The meaning and use of the area under a receiver operating characteristic (ROC) curve. Radiology. 1982;143(1):29–36. doi: 10.1148/radiology.143.1.7063747 7063747 10.1148/radiology.143.1.7063747

[17] Dalegrave D, Silva RL, Becker M, Gehrke LV, Friedman G. Relative adrenal insufficiency as a predictor of disease severity and mortality in severe septic shock. Rev Bras Ter Intensiva. 2012;24(4):362–8. doi: 10.1590/S0103-507X2012000400012 23917934 10.1590/S0103-507X2012000400012 PMC4031805

[18] Rai SS, O'Connor SN, Lange K, Rivett J, Chapman MJ. Enteral nutrition for patients in septic shock: a retrospective cohort study. Crit Care Resusc. 2010;12(3):177–81. 21261575

[19] Omar S, Burchard AT, Lundgren AC, Mathivha LR, Dulhunty JM. The relationship between blood lactate and survival following the use of adrenaline in the treatment of septic shock. Anaesth Intensive Care. 2011;39(3):449–55. 21675065 10.1177/0310057X1103900316

[20] McEvoy C, Kollef MH. Determinants of hospital mortality among patients with sepsis or septic shock receiving appropriate antibiotic treatment. Curr Infect Dis Rep. 2013;15(5):400–6. doi: 10.1007/s11908-013-0361-1 23975687 10.1007/s11908-013-0361-1

[21] Labelle A, Juang P, Reichley R, Micek S, Hoffmann J, Hoban A, et al The determinants of hospital mortality among patients with septic shock receiving appropriate initial antibiotic treatment*. Crit Care Med. 2012;40(7):2016–21. doi: 10.1097/CCM.0b013e318250aa72 22584765 10.1097/CCM.0b013e318250aa72

[22] Pavon A, Binquet C, Kara F, Martinet O, Ganster F, Navellou JC, et al Profile of the risk of death after septic shock in the present era: an epidemiologic study. Crit Care Med. 2013;41(11):2600–9. doi: 10.1097/CCM.0b013e31829a6e89 23963127 10.1097/CCM.0b013e31829a6e89

[23] Singer M, Deutschman CS, Seymour CW, Shankar-Hari M, Annane D, Bauer M, et al The Third International Consensus Definitions for Sepsis and Septic Shock (Sepsis-3). JAMA. 2016;315(8):801–10. doi: 10.1001/jama.2016.0287 26903338 10.1001/jama.2016.0287 PMC4968574

[24] Bai X, Yu W, Ji W, Lin Z, Tan S, Duan K, et al Early versus delayed administration of norepinephrine in patients with septic shock. Crit Care. 2014;18(5):532 doi: 10.1186/s13054-014-0532-y 25277635 10.1186/s13054-014-0532-y PMC4194405

[25] Morimatsu H, Singh K, Uchino S, Bellomo R, Hart G. Early and exclusive use of norepinephrine in septic shock. Resuscitation. 2004;62(2):249–54. doi: 10.1016/j.resuscitation.2004.03.016 15294412 10.1016/j.resuscitation.2004.03.016

[26] Cecconi M, Arulkumaran N, Kilic J, Ebm C, Rhodes A. Update on hemodynamic monitoring and management in septic patients. Minerva Anestesiol. 2014;80(6):701–11. 24280808

[27] Kato R, Pinsky MR. Personalizing blood pressure management in septic shock. Ann Intensive Care. 2015;5(1):41 doi: 10.1186/s13613-015-0085-5 26573630 10.1186/s13613-015-0085-5 PMC4646890

[28] Marik P, Bellomo R. A rational approach to fluid therapy in sepsis. Br J Anaesth. 2016;116(3):339–49. doi: 10.1093/bja/aev349 26507493 10.1093/bja/aev349

[29] Loubani OM, Green RS. A systematic review of extravasation and local tissue injury from administration of vasopressors through peripheral intravenous catheters and central venous catheters. J Crit Care. 2015;30(3):653 e9–17. 10.1016/j.jcrc.2015.01.014 25669592

[30] Lampin ME, Rousseaux J, Botte A, Sadik A, Cremer R, Leclerc F. Noradrenaline use for septic shock in children: doses, routes of administration and complications. Acta Paediatr. 2012;101(9):e426–30. doi: 10.1111/j.1651-2227.2012.02725.x 22568565 10.1111/j.1651-2227.2012.02725.x

[31] Turner DA, Kleinman ME. The use of vasoactive agents via peripheral intravenous access during transport of critically III infants and children. Pediatr Emerg Care. 2010;26(8):563–6. doi: 10.1097/PEC.0b013e3181ea71e1 20657339 10.1097/PEC.0b013e3181ea71e1

[32] Hodzic S, Golic D, Smajic J, Sijercic S, Umihanic S. Complications Related to Insertion and Use of Central Venous Catheters (CVC). Med Arch. 2014;68(5):300–3. doi: 10.5455/medarh.2014.68.300-303 25568558 10.5455/medarh.2014.68.300-303 PMC4276017

[33] Ricard JD, Salomon L, Boyer A, Thiery G, Meybeck A, Roy C, et al Central or peripheral catheters for initial venous access of ICU patients: a randomized controlled trial. Crit Care Med. 2013;41(9):2108–15. doi: 10.1097/CCM.0b013e31828a42c5 23782969 10.1097/CCM.0b013e31828a42c5

