# Supplementary material for: Septic shock and the use of norepinephrine in an intermediate care unit: Mortality and adverse events
Source: PLoS One. 2017 Aug 24;12(8):e0183073. doi: 10.1371/journal.pone.0183073 (PMC5570296; doi:10.1371/journal.pone.0183073)
Supplement: S2 File — (PDF) [file pone.0183073.s002.pdf]

Karolinska Institutet

Letter explaining "Bench Fee"
June 1st 2017

Ioannis P Androulakis
Academic Editor
PLOS ONE

Dear Ioannis P Androulakis,

During the autumn term of 2013 Mikael Hallengren, medical student at Karolinska Institutet, completed the compulsory master thesis course (2LK028). During this course a scientific paper is written at a department or clinic. Karolinska Institutet pays a bench fee to the departments (23.000 SEK) as a coverage for the expenses (e.g computer programs, travels, laboratory reagents etc) to receive and supervise a student. Thus, there is no grant name, grant number or URL. In this particular example the department KIDS (Karolinska Institutet at Danderyd Hospital) received the bench fee.

Best regards,

Jeanette Danielsson
Education Officer
Medical program
Karolinska Institutet

Karolinska Institutet

Postadress
Box 281
171 77 Stockholm
Org.nummer 202100 2973

Besöksadress
Nobels väg 12
Solna

Telefon
08-5248 0000

Fax
08-31 49 75

Webb
www.meb.ki.se
